# Novel anticarcinoembryonic antigen antibody–drug conjugate has antitumor activity in the existence of soluble antigen

**DOI:** 10.1002/cam4.1003

**Published:** 2017-02-17

**Authors:** Daisuke Shinmi, Ryosuke Nakano, Keisuke Mitamura, Minami Suzuki‐Imaizumi, Junko Iwano, Yuya Isoda, Junichi Enokizono, Yasuhisa Shiraishi, Emi Arakawa, Kazuma Tomizuka, Kazuhiro Masuda

**Affiliations:** ^1^ Research Core Function LaboratoriesResearch Functions UnitR&D DivisionKyowa Hakko Kirin Co., Ltd.TokyoJapan; ^2^ Oncology Research LaboratoriesOncology R&D UnitR&D DivisionKyowa Hakko Kirin Co., Ltd.TokyoJapan; ^3^ Translational Research UnitR&D DivisionKyowa Hakko Kirin Co., Ltd.TokyoJapan; ^4^ Innovative Technology LaboratoriesResearch Functions UnitR&D DivisionKyowa Hakko Kirin Co., Ltd.TokyoJapan; ^5^ R&D Planning DepartmentR&D DivisionKyowa Hakko Kirin Co., Ltd.TokyoJapan; ^6^ Fuji Research ParkR&D DivisionKyowa Hakko Kirin Co., Ltd.TokyoJapan

**Keywords:** Antibody, antibody–drug conjugate, CEA, esophageal cancer, gastric cancer

## Abstract

Carcinoembryonic antigen (CEA) is a classic tumor‐specific antigen that is overexpressed in several cancers, including gastric cancer. Although some anti‐CEA antibodies have been tested, to the best of our knowledge, there are currently no clinically approved anti‐CEA antibody therapies. Because of this, we have created the novel anti‐CEA antibody, 15‐1‐32, which exhibits stronger binding to membrane‐bound CEA on cancer cells than existing anti‐CEA antibodies. 15‐1‐32 also shows poor affinity for soluble CEA; thus, the binding activity of 15‐1‐32 to membrane‐bound CEA is not influenced by soluble CEA. In addition, we constructed a 15‐1‐32‐monomethyl auristatin E conjugate (15‐1‐32‐vcMMAE) to improve the therapeutic efficacy of 15‐1‐32. 15‐1‐32‐vcMMAE showed enhanced antitumor activity against gastric cancer cell lines. Unlike with existing anti‐CEA antibody therapies, antitumor activity of 15‐1‐32‐vcMMAE was retained in the presence of high concentrations of soluble CEA.

## Introduction

Monoclonal antibodies are major therapeutic agents used to treat many types of cancer, as well as immunological and other diseases. Currently, more than 40 therapeutic antibodies are in the market. Despite the clinical success of these antibodies, many clinical trials have failed to show a benefit in patients owing to lack of efficacy. Fc engineering and antibody–drug conjugate (ADC) are emerging technologies designed to improve the efficacy of antibody therapies 1, 2, 3. In addition to new technologies, discovery of antibodies that show cancer‐specific binding properties is required, because many antibodies are discarded during clinical trial after being shown to be toxic to normal tissue.

Carcinoembryonic antigen (CEA or CEACAM5) is a 180‐kDa glycosylated protein that is expressed on the surface membrane of many types of cancer cells and is widely used as a diagnostic marker for various cancers, including colorectal, gastric, and liver cancer 4, 5, 6. Thus, CEA is an attractive target for cancer therapy, especially for antibody therapy. Currently, there are several anti‐CEA antibodies in clinical trial; however, no CEA‐targeted antibody has been marketed so far 7, 8, 9, 10. There are two major problems for cancer immunotherapy using CEA‐targeted antibodies. First, the functional significance of CEA in tumorigenesis is thought to be limited because CEA is glycophosphatidylinositol (GPI) anchor protein and has no intracellular domain, which is required for signal transduction. Many anti‐CEA antibodies have been reported; however, there are few reports of anti‐CEA antibodies that inhibit tumor activity. Second, CEA is cleaved from the cell surface in a soluble form 11. This property of CEA is useful as a clinical marker 12, 13 but not as the target of a therapeutic antibody. Because highly secreted soluble CEA around the tumor and in serum may disturb the therapeutic antibody from binding to membrane‐form CEA, accumulation of therapeutic anti‐CEA antibody in the tumor is limited.

ADC is an established antibody therapeutics format that covalently links a cytotoxic agent to antibodies that recognize tumor antigens to enhance the antitumor potency of antibody therapies 14, 15. Antibodies which are conjugated with the cytotoxic agent bind to tumor tissue expressing the tumor antigen, get internalized into the intracellular space, and then release the cytotoxic agent inside the cell. Two ADCs are already on the market, including CD30‐targeted brentuximab vedotin for relapsed Hodgkin's lymphoma and anaplastic large cell lymphoma 16, and Her2‐targeted trastuzumab emtansine for refractory Her2‐expressing breast cancers 17. More than 40 ADCs are in clinical trial including an anti‐CEA ADC, IMMU‐130 (labetuzumab‐SN‐38), which is now in phase II clinical trials for colorectal cancer 7.

15‐1‐32 is a fully human antibody that recognizes membrane‐bound CEA. 15‐1‐32 shows a higher binding affinity for membrane‐bound CEA than other CEA antibodies, and is only slightly influenced by soluble‐form CEA. Another group also reported that the anti‐CEA antibody, PR1A3, binds only membrane‐bound CEA 18, 19, 20, 21, but our antibody recognizes a different epitope and displays a much higher binding activity to the membrane‐bound CEA. Despite the unique binding property, 15‐1‐32 retains the reactivity to the tumor tissue compared to existing CEA antibodies. In this study, we constructed 15‐1‐32‐drug conjugate (15‐1‐32‐vcMMAE) to improve the therapeutic efficacy of 15‐1‐32 and investigated the antitumor effects.

## Materials and Methods

### Materials

Maleimidocaproyl‐valine‐citrulline‐p‐aminobenzyloxycarbonyl‐monomethyl auristatin E (vcMMAE) was obtained from MedChem Express (Princeton, NJ). Alexa Fluor 488 (Alexa488) C5 Maleimide was purchased from Thermo Fisher Scientific (Waltham, MA). Rituximab and anti‐2, 4‐dinitrophenol (DNP) antibody were internally produced. Human soluble CEA was purchased from HyTest (Turku, Finland). L929 mouse fibroblast cell line was obtained from Riken Cell Bank (Tsukuba, Japan) and cultured at 37°C with 5% carbon dioxide (CO_2_) in Minimum Essential Medium supplemented with 10% FBS. Expi293F Expression System was purchased from Thermo Fisher Scientific (Waltham, MA). MKN‐45 human gastric cancer cell line was obtained from Japanese Collection of Research Bioresources (Osaka, Japan) and cultured at 37°C with 5% CO_2_ in RPMI1640 supplemented with 10% FBS. KATO‐III human gastric cancer cell line was obtained from Sumitomo Dainippon Pharma (Osaka, Japan) and cultured at 37°C with 5% CO_2_ in RPMI1640 supplemented with 10% FBS. Raji human Burkitt's lymphoma cell line was obtained from American Type Culture Collection (Manassas, VA) and cultured at 37°C with 5% CO_2_ in RPMI1640 supplemented with 10% FBS. LS174T human colon cancer cell line was obtained from American Type Culture Collection (Manassas, VA) and cultured at 37°C with 5% CO_2_ in MEM supplemented with 10% FBS. SK‐BR‐3 human breast cancer cell line was obtained from American Type Culture Collection and cultured at 37°C with 5% CO_2_ in McCoy's 5A supplemented with 10% FBS. Frozen human tumor samples were purchased from Tissue Solutions Ltd. (Glasgow, UK).

### Animals

KM mice were obtained from an in‐house breeding facility 3. C.B17/Icr‐scid Jcl (SCID) mice were purchased from Clea (Tokyo, Japan). All animal studies were performed in accordance with Standards for Proper Conduct of Animal Experiments at Kyowa Hakko Kirin Co., Ltd. under the approval of the company's Institutional Animal Care and Use Committee. Tokyo Research Park of Kyowa Hakko Kirin Co., Ltd. is fully accredited by the Association for the Assessment and Accreditation of Laboratory Animal Care, International.

### Generation of transfected cells

Human CEA (GenBank accession No. NM_004363.5) was generated by PCR and cloned into the INPEP4 (Biogen IDEC, Inc., Cambridge, MA) vector. Human CA9 (GenBank accession No. NM_001216.2) cDNA was cloned into the pKANTEX93 vector. Each expression vector was introduced into L929 cells via electroporation to obtain G418‐resistant clones.

### Isolation of the anti‐CEA mAb

KM mice were i.p. immunized with CEA‐expressing L929 transfectant (1 × 10^7^ cells per head per shot) weekly for 5 weeks. Spleen cells were fused with SP2/0 cells (American Type Culture Collection). Antibody‐secreting hybridomas were initially screened by flow cytometry, as described below, with CEA‐expressing L929 transfectants as positive selection and CEA‐null L929 transfectants as negative selection. Hybridomas were further selected by competitive flow cytometry with CEA‐expressing L929 transfectants and soluble CEA. We selected clone 15‐1‐32 based upon the results of flow cytometry and carried out limiting dilution to isolate a single clone, producing a monoclonal antibody.

### Antibody and conjugate generation

The heavy‐ and light‐chain variable region cDNAs from the hybridoma cells producing 15‐1‐32 were isolated by PCR and cloned into the pKANTEX93 vector for production of the recombinant 15‐1‐32 antibody with mammalian cells 22. Existing anti‐CEA antibodies, PR1A3 and labetuzumab, were also cloned into the mammalian expression vectors. Each anti‐CEA antibody (15‐1‐32, PR1A3, and labetuzumab) was stably expressed in DG44 cells and these transfectants were cultured for about 1 week. The culture supernatant was applied onto 1 mL MabSelect SuRe Protein A resin (GE Healthcare, Piscataway, NJ). The resin was washed with 20 mL of D‐PBS. The bound antibody was eluted with 5 mL of 100 mmol/L glycine‐HCl (pH 3.5) and neutralized using 1 mol/L Tris‐HCl (pH 8.0). The buffer exchange into 20 mmol/L Citrate and 150 mmol/L sodium chloride (NaCl) (pH6.0) was accomplished using Amicon Ultra 4 device (Merck Millipore, Billerica, MA). ADC format of 15‐1‐32 and labetuzumab were genetically spliced into N5KG1‐Val Lark vector (Biogen IDEC, Inc., Cambridge, MA), expressed with Expi293F Expression System, and purified as described above. Antibody–vcMMAE conjugate and Alexa488 conjugate were prepared as described previously 23.

### Binding analysis by flow cytometry

The specificity of 15‐1‐32 against CEA was determined by flow cytometry. Adherent MKN‐45 and KATO‐III cells were detached from the dish with Cell Dissociation Buffer (ThermoFisher), suspended in 96‐well round‐bottomed plates at a density of 2  ×  10^5^ cells/well, and washed with FACS buffer (1% BSA, 2 mmol/L EDTA, and 0.05% sodium azide in D‐PBS). Floating Raji cells were collected in 96‐well round‐bottomed plates in the same way. 15‐1‐32, labetuzumab, 12‐140‐1 (mouse anti‐human CEA antibody, LifeSpan BioSciences, Seattle, WA), rituximab, and human IgG1 (Sigma‐Aldrich, St. Louis, MO) were added to wells at concentrations of 1 *μ*g/mL, and then incubated at 4°C for 1 h. After washing, secondary antibody (Alexa Fluor 488 Goat Anti‐human or mouse IgG (H+L) Antibody, Thermo Fisher Scientific, Waltham, MA) was added to each well (1: 2000 diluted in FACS buffer) and incubated at 4°C for 1 h. After washing, the cells were analyzed using a FACSVerse System (BD Biosciences PharMingen). The competitive analysis was conducted as follows. Alexa488‐labeled 15‐1‐32 was added to MKN‐45 cells at concentrations of 800 ng/mL, then incubated with the same volume of 12‐140‐1 PR1A3, labetuzumab, and human IgG1 at concentrations of 20, 2, 0.2, or 0.02 *μ*g/mL at 4°C for 1 h. After washing, the cells were analyzed using a FACSVerse System.

The binding activity of anti‐CEA antibody with or without soluble CEA was also determined by flow cytometry. MKN‐45 cells were detached from the dish with Cell Dissociation Buffer (ThermoFisher), suspended in 96‐well round‐bottomed plates at a density of 2  ×  10^5^ cells/well, and washed with FACS buffer. 15‐1‐32, PR1A3, labetuzumab, and human IgG1 as a negative control, were added to wells at concentrations of 80 ng/mL, then incubated with the same volume of human soluble CEA at concentrations of 200, 20, or 2 μg/mL at 4°C for 1 h. After washing, secondary antibody was added to each well and incubated at 4°C for 1 h. After washing, the cells were analyzed using a FACSVerse System.

### Internalization analysis of 15‐1‐32

The internalization of anti‐CEA antibody was determined by modified method as described previously 24. Firstly, the quantitative determination of cell surface antigens on MKN‐45 and KATO‐III were conducted. 12‐140‐1 was added to the cells which collected in the same way as described above at concentrations of 100 nmol/L, and then incubated on ice for 1 h. After washing with PBS‐0.1% BSA‐Azide (0.1% BSA and 0.1% sodium azide in D‐PBS), FITC‐labeled anti‐mouse IgG F(ab’)_2_ was added to each well and incubated on ice for 1 h. Quantification of cell surface CEA expression was done with QIFIKIT (DAKO A/S, Glostrop, Denmark) according to the manufacturer's protocol. Next, internalization of 15‐1‐32 was evaluated by a quenching method. Alexa488‐labeled 15‐1‐32 was added to the cells at concentrations of 150 nM and incubated at 37°C with 5% CO_2_ for 0.5, 1, 2, 4, 6, and 24 h. The antibody solution was removed and the cells were washed with ice‐cold PBS‐Azide (0.1% sodium azide in D‐PBS). Then quenching antibody (Anti‐Alexa Flour 488, Rabbit IgG, Thermo Fisher Scientific) was added to wells and left on ice for 1 h (Quenched cells). For unquenched sample, PBS‐Azide was added instead of the quenching antibody solution (Unquenched cells). Cells were incubated for 1 h on ice in Alexa Fluor 488‐labeled 15‐1‐32 followed by incubation for 1 h on ice in PBS‐Azide (initial cells). Cells were incubated for 1 h on ice in the Alexa Fluor 488‐labeled 15‐1‐32 followed by incubation for 1 h on ice with the quenching antibody solution (Quenched initial cells). After removing the quenching antibody solution or PBS‐Azide, the cells were collected and analyzed by the flow cytometer. Nontreated cells were analyzed as blank cells.

MFI of samples were measured by flow cytometer. Internalized ratio (% initial) of 15‐1‐32 was calculated by the following equation: Internalized ratio(%initial)= (MFI of Quenched cells−MFI of Quenched initial cells)×100/ (MFI of initial cells−MFI of blank cells).Internalized number of antibody was obtained by multiplying internalized ratio to number of antibodies bound to cell surface.

### Kinetic Analysis of the binding property of anti‐CEA antibodies by Biacore

Binding affinity and kinetics analyses were conducted using the Biacore T100 and Human Antibody Capture Kit (GE Healthcare, Piscataway, NJ). The murine anti‐human IgG antibody was immobilized on a CM5 sensor chip according to the instruction manual. 15‐1‐32 (1.25 *μ*g/mL) or labetuzumab in HBS‐EP+ running buffer (10 mmol/L HEPES, pH 7.4, 150 mmol/L NaCl, 3 mmol/L EDTA, 0.005% surfactant P20) was captured for 40 sec at a flow rate of 10 *μ*L/min. A range of concentrations of human soluble CEA (0, 0.14, 0.41, 1.2, 3.7, 11, 33, and 100 nmol/L) in HBS‐EP+ running buffer were passed over the captured 15‐1‐32 or labetuzumab for 180 sec at a flow rate of 30 μL/min during the association phase. At the dissociation phases, HBS‐EP+ running buffer was exposed for 600 sec at a flow rate of 30 *μ*L/min. Chip regeneration was accomplished by exposure to 3 mol/L magnesium chloride for 30 sec at a flow rate of 30 *μ*L/min. All kinetic measurements were conducted at 25°C. Binding kinetic parameters, including the kass, kdiss, and KD values, were calculated using Biacore evaluation software.

### Immunohistochemistry

Frozen samples of 10 stomach adenocarcinomas and 10 esophageal squamous cell carcinomas were used as test tissue. MKN‐45 and LS174T mouse xenograft were used as the positive control tissue and L929‐CA9 mouse xenograft was used as the negative control tissue. All tumor samples and xenografts were sectioned at 6 *μ*m, allowed to air‐dry for 1 h, fixed in acetone for 10 min at room temperature and stored at −80°C. Slides were air‐dried and treated with sodium azide (1 mmol/L), glucose (10 mmol/L), and glucose oxidase (2 U/mL) for 60 min at 37°C. Following a wash in PBS (pH 7.2), slides were blocked with avidin and biotin solution (DAKO) for 20 min each and blocked with 1% BSA for 10 min. The primary antibodies, 15‐1‐32, labetuzumab, and anti‐DNP antibody (10 *μ*g/mL each), were applied for 1 h at room temperature 25. Excess primary antibodies were washed off and sections were covered with streptavidin‐HRP (NICHIREI BIOSCIENCE INC., Tokyo Japan) for 30 min, after which 3,3′‐diaminobenzidine (DAB) was applied for 4 min. Slides were counterstained with hematoxylin, dehydrated, and coverslipped for evaluation under a light microscope. Slides were graded according to the following criteria and sites of positive staining were recorded. Staining in cells or tissue was judged as significant when staining intensity in the test antibodies exceeded that in the control antibodies at the equivalent concentration.

Staining intensity: ‐, negative; +, faint/light; 2 + , light‐medium; 3 + , moderate; 4 + , dark.

Site of staining: M, membranous; C, cytoplasmic.

Staining frequency: Negative, no labeled cell; Very rare, <25%; Rare, >= 25% and <50%; Occasional, >= 50% and <75%; Frequent, >= 75%.

### Proliferation assays

MKN‐45 was seeded at a density of 3 × 10^3^ cells/well in clear bottom, white wall, 96‐well plates (Greiner Bio‐One, Kremsmünster, Austria). After 24 h, dilution of anti‐CEA antibody vcMMAE conjugates were added to cells at final concentrations of 10,000, 3000, 1000, 300, 100, 30, 10, and 3 ng/mL and incubated at 37°C for 120 h. Cell viability was then determined using the Cell Titer‐Glo luminescent assay (Promega Corp, Madison, WI) following the manufacturer's instructions.

## Results

### Isolation of an anti‐CEA antibody, 15‐1‐32

**Figure 1:**
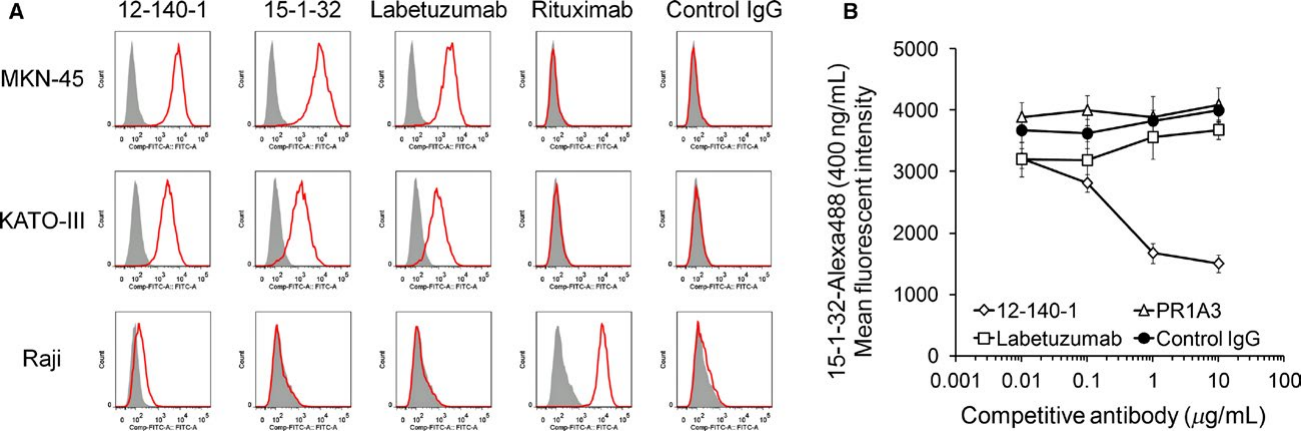
(A) Histograms of antibody binding to carcinoembryonic antigen (CEA) expressing MKN‐45 and KATO‐III, CEA negative Raji cells by flow cytometry (red histograms). The secondary antibody only served as controls (gray‐filled histograms). (B) Competitive flow cytometric analysis with Alexa488‐labeled 15‐1‐32 against CEA‐expressing MKN‐45 cells. 12‐140‐1 (open rhombus), labetuzumab (open square), and PR1A3 (open triangle) were used as competitors.

We first aimed to identify anti‐CEA antibodies with high affinity for only membrane‐bound CEA. To isolate anti‐CEA antibodies, KM mice were immunized with CEA‐expressing L929 transfectant 3. Hybridoma cells produced from spleen cells were selected using flow cytometry. One of the anti‐CEA antibodies obtained was 15‐1‐32, a fully human anti‐CEA antibody, which bound to CEA expressing MKN‐45 and KATO‐III cells in the same manner as labetuzumab and mouse anti‐human CEA antibody, 12‐140‐1, which is commercially available but did not bind to CEA negative Raji cells. (Fig. 1A) These results indicated that 15‐1‐32 displayed strong and specific binding to membrane‐bound CEA. Another group has reported that the anti‐CEA antibody, PR1A3, binds only membrane‐bound CEA. PR1A3 recognizes the B3 domain located at the C‐terminal of CEA 19. For exploration of a 15‐1‐32‐binding epitope, we conducted competition experiments against existing anti‐CEA antibodies, including PR1A3, labetuzumab, and other anti‐CEA antibodies, using flow cytometry analysis (Fig. 1B). PR1A3 did not inhibit 15‐1‐32 binding, but 12‐140‐1, which recognizes the N‐terminal domain of CEA 26, inhibited 15‐1‐32 binding. These results suggest that 15‐1‐32 targets the N‐terminal domain of CEA and that its recognition epitope is different from that of PR1A3.

### Binding property of 15‐1‐32

**Figure 2:**
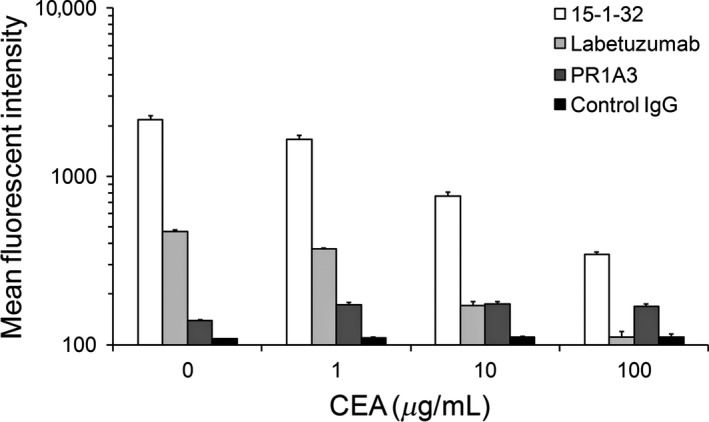
Binding activity of anti‐carcinoembryonic antigen (CEA) antibodies to membrane‐bound CEA on MKN‐45 under the presence or absence of soluble CEA.

Next, we validated the binding activity of 15‐1‐32 to membrane‐bound CEA, compared to PR1A3 and labetuzumab, with or without soluble CEA, by flow cytometry analysis. As shown in Figure 2, 15‐1‐32 showed a much higher binding affinity to membrane‐bound CEA expressed on MKN‐45 than the other two anti‐CEA antibodies. In the presence of soluble CEA, labetuzumab had a reduced binding activity to cell surface CEA. However, there was only a slight reduction in the reactivity of PR1A3 and 15‐1‐32 with MKN‐45, even in the presence of 10 μg/mL soluble CEA. The reactivity of 15‐1‐32 with MKN‐45 is almost 10 times higher than PR1A3 in this condition.

The binding kinetics of 15‐1‐32, PR1A3, and labetuzumab to soluble CEA were analyzed on a BIAcore biosensor in order to clarify the mechanism of binding of 15‐1‐32. The sensorgrams and the kinetic parameters for antibody‐soluble CEA interactions are shown in Figure S1 and Table 1, respectively. The correct kinetic parameters of PR1A3 could not be calculated because of its weak reactivity with soluble CEA. Equilibrium dissociation constant (KD) of 15‐1‐32 had only an approximately fourfold difference compared with labetuzumab; however, the binding properties of 15‐1‐32 are significantly different from those of labetuzumab. 15‐1‐32 had an approximately threefold higher association rate constant (kass) for soluble CEA than labetuzumab, whereas 15‐1‐32 had an approximately 12‐fold higher dissociation constant (kdiss). These results indicated that 15‐1‐32 showed week binding property against soluble CEA.

**Table 1:** Binding kinetics of anti‐CEA antibody against soluble CEA

Antibody	*k*_ass_ (x 10^4^) M^−1^ S^−1^	*k*_diss_ (× 10^−4^) S^−1^	*K*_D_ (*k*_d_/*k*_a_) nM
15‐1‐32	15.6	104	66.3
Labetuzumab	5.08	8.38	16.5

### Internalization property of 15‐1‐32

**Figure 3:**
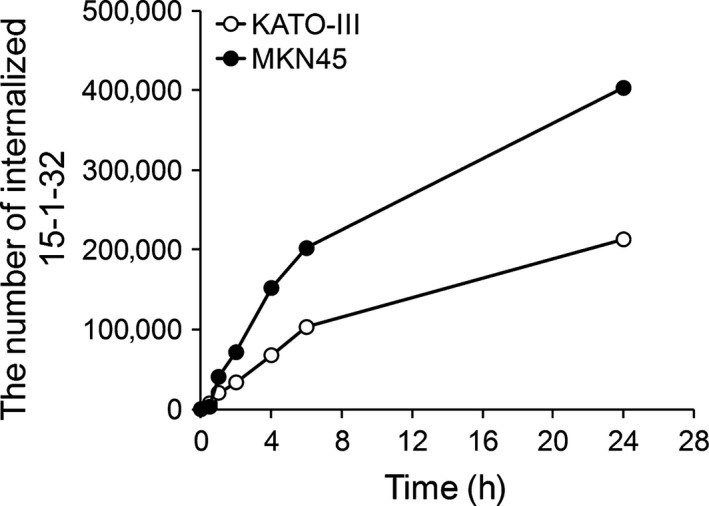
The time‐dependent change of the number of internalized 15‐1‐32 antibody into KATO‐III (open circle) and MKN‐45 (closed circle).

The internalization property of 15‐1‐32 on each cell line was evaluated by flow cytometry analysis. The number of internalized 15‐1‐32 which bound to membrane‐bound CEA in each of the time was calculated and shown in Figure 3. About 400,000 antibodies were internalized into MKN‐45 cells for 24 h. Although the number of internalized 15‐1‐32 in KATO‐III were less than that in MKN‐45, more than 200,000 molecules were internalized into each cell. These results indicated that 15‐1‐32 efficiently internalizes into CEA expressing cell depend on antigen‐antibody binding and is applicable to ADC technology.

### Reactivity of 15‐1‐32 to the tumor tissue

**Figure 4:**
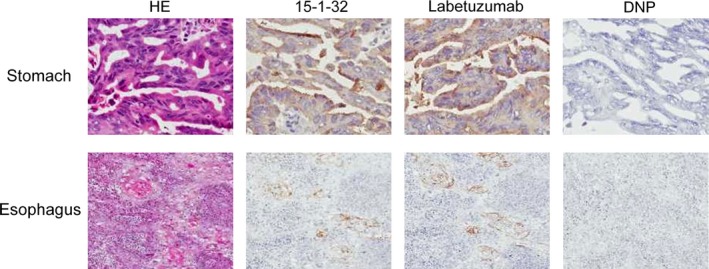
Immunhistochemical staining of carcinoembryonic antigen (CEA) with 15‐1‐32 and labetuzumab. Sections from tumoral stomach adenocarcinoma tissues with an antibody against CEA are shown in the upper panels. Lower panels show esophagus squamous cell carcinoma sections. Anti‐DNP was used as negative control. HE stands for hematoxylin‐eosin.

To examine the reactivity of 15‐1‐32 to human gastric cancer tissue by immunohistochemistry, we evaluated 10 surgical specimens to confirm CEA expression in gastric (Fig. 4 upper) and esophageal cancer (Fig. 4 lower). Despite the unique binding property of 15‐1‐32 to soluble CEA, positive staining was observed in 10 out of 10 gastric cancer tissue samples and six out of 10 esophageal cancer tissue samples, each stained with both test antibodies (Table 2). The sites of staining were in the membrane and/or the cytoplasm of the tumor cells. Localization of positive staining was apical in adenocarcinoma and squamous cell carcinoma. The frequency of staining was varied in gastric cancer tissue (less than 25% to more than 75%) and less than 25% in esophageal cancer tissue. In some esophageal cancer samples, normal esophageal epithelium (mainly apical) also showed positive staining. There was a similar trend with respect to the staining site, intensity, and frequency between 15‐1‐32 and labetuzumab.

**Table 2:** Cross‐reactivity of anti‐CEA antibodies on human stomach and esophagus tumors

Frozen human tissue								15‐1‐32			Labetuzumab			Anti‐DNP		
NO.	Sex	Age	Organ	Diagnosis	Grade	TNM	Stage	Site	Intensity	Frequency	Site	Intensity	Frequency	Site	Intensity	Frequency
			MKN45					MC	2+	4	CM	+	4		–	
			LS174T					MC	+	1	C	+	4		–	
			L929‐CA9						−			−			–	
081293T2(1)	F	72	Stomach	Adenocarcinoma	G3	T4bN0M0	IIIB	C	2+	4	C	2+	4		–	
081275T2(2)	F	77	Stomach	Adenocarcinoma	G2	T4bN0M0	IIIB	MC	+/2+^a^	4	MC	+/2+^a^	4		–	
081274T2(1)	M	66	Stomach	Adenocarcinoma	G1	T2N0M0	IB	MC	+/2+^a^	2	MC	+/2+^a^	2		–	
081271T2(2)	F	63	Stomach	Adenocarcinoma	G4	T3N0M0	IIA	C	2+	1	C	2+	1		–	
081202T2(4)	F	67	Stomach	Adenocarcinoma	G3	T3N3bM0	IIIB	C	2+	2	C	2+	2		–	
081200T2(3)	M	70	Stomach	Adenocarcinoma	G2	T3N2M0	IIIA	MC	+/2+^a^	4	MC	+/2+^a^	4		–	
081198T2(2)	M	77	Stomach	Adenocarcinoma	G1‐2	T1bN0M0	IA	MC	+	1	MC	2+	1		–	
081196T2(1)	M	62	Stomach	Adenocarcinoma	G3	T3N2M1	IV	MC	2+	1	MC	2+	1		–	
081193T2(1)	F	62	Stomach	Adenocarcinoma	G2	T3N0M0	IIA	MC	+/2+^a^	4	MC	+/2+^a^	4		–	
081188T2(1)	M	61	Stomach	Adenocarcinoma	G3	T3N3aM0	IIIB	C^b^	+	1	MC	+	1		–	
21538T2(2)	M	71	Esophagus	Squamous cell carcinoma	G3	T3N1M0	IIIA	MC^b^	2+	1	MC^b^	2+	1		–	
21537T2(2)	M	65	Esophagus	Squamous cell carcinoma	G2	T3N2M0	IIIB	MC^b^	2+	1	MC^b^	2+	1		–	
21534T2(1)	M	52	Esophagus	Squamous cell carcinoma	G3	T3N0M0	IIA	C^b^	2+	1	C^b^	2+	1		–	
21513T2(2)	M	57	Esophagus	Squamous cell carcinoma	G3	T3N2M0	IIIB	C^b^	2+	2	C^b^	2+	1		–	
21497T2(2)	M	71	Esophagus	Squamous cell carcinoma	G3	T3N2M0	IIIB	MC^b^	+	1	MC^b^	+	1		–	
21492T2(3)	M	51	Esophagus	Squamous cell carcinoma	G2	T4aN1M0	IIIC		−			−			–	
21491T2(1)	M	55	Esophagus	Squamous cell carcinoma	G2	T3N1M0	IIIA		−			−			–	
21489T2(2)	M	55	Esophagus	Squamous cell carcinoma	G2	T2N1M0	IIB	MC^b^	2+	2	C^b^	2+	2		–	
21486T2(1)	M	51	Esophagus	Squamous cell carcinoma	G2	T1bN0M0	IB		−			−			–	
21474T2(3)	M	57	Esophagus	Squamous cell carcinoma	G3	T3N1M0	IIIA		−			−			–	

Site or description of staining; M: membranous, C: cytoplasmic. NA: not applicable.

Criteria of staining intensity; ‐: negative, 1 + : faint/light, 2 + : light‐medium, 3 + : moderate, 4 + : dark.

Criteria of staining frequency; ‐ (no labeled cells “‐ative”), 1:<25%, 2:>=25% and <50%, 3:>=50% and <75%, 4:>=75%.

a Apical: 2 + , other: +.

b Positive staining was mainly observed in apical.

### The effects of 15‐1‐32‐vcMMAE on CEA‐expressing cells in vitro

**Figure 5:**
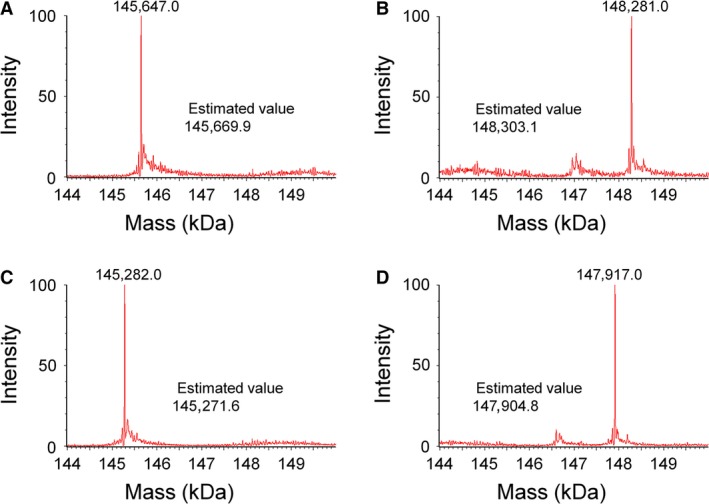
Deconvoluted mass spectra of naked 15‐1‐32 Actibody (A), 15‐1‐32‐vcMMAE (B), naked labetuzumab Actibody (C), and labetuzumab‐vcMMAE (D). The most intense peak in each mass spectrum corresponds to the molecular weight of the antibody.

To enhance the antitumor potency of 15‐1‐32, we constructed an ADC. Currently, there are two major tubulin inhibitors developed as ADC payloads, auristatins and maytansine derivatives 27. We chose the auristatin derivative, vcMMAE, to conjugate with 15‐1‐32. We applied a novel site‐specific conjugation technology, Actibody, to control the number of attached drug molecules to cysteine residues in the antibody via thiol‐reactive molecules, such as maleimide 23. As a result of conjugation, the ADC, termed 15‐1‐32‐vcMMAE, contains two molecules of vcMMAE per antibody (Fig. 5A, B). We also constructed a control ADC, termed labetuzumab‐vcMMAE, with Actibody technology (Fig. 5C, D).

**Figure 6:**
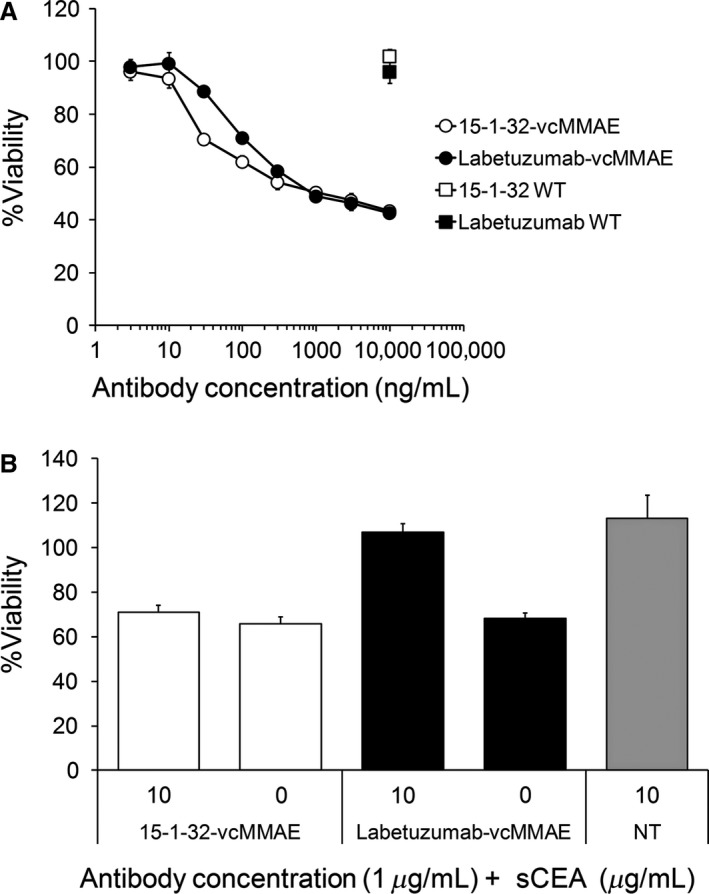
(A) In vitro cytotoxicity of 15‐1‐32‐vcMMAE (open circle), labetuzumab‐vcMMAE (closed circle), naked 15‐1‐32 (open square), naked labetuzumab (closed square) on carcinoembryonic antigen (CEA)‐positive gastric cancer cell lines, MKN‐45. (B) Effect of soluble CEA blocking on the cytotoxicity of 15‐1‐32‐vcMMAE and labetuzumab‐vcMMAE, against MKN‐45 cells. NT stands for not antibody treated.

15‐1‐32‐vcMMAE exerted cytotoxic activity against CEA‐expressing MKN‐45 cells, which led to the death of 60% of all target cells at 10 μg/mL (Fig. 6A). Cytotoxic activity of 15‐1‐32‐vcMMAE was almost the same as labetuzumab‐vcMMAE. To further confirm the influence of soluble CEA, we evaluated the cytotoxicity of 15‐1‐32‐vcMMAE and labetuzumab‐vcMMAE in the presence of soluble CEA (Fig. 6B). The cytotoxic activity of labetuzumab‐vcMMAE was almost canceled in the presence of soluble CEA. On the other hand, 15‐1‐32‐vcMMAE retained antiproliferative activity against the MKN‐45 cells, despite the existence of soluble CEA.

## Discussion

CEA has been reported to be overexpressed in various solid tumors. Although many anti‐CEA antibodies have been created and developed since this molecule was discovered, to the best of our knowledge, no anti‐CEA antibodies have been approved for clinical use. The functional correlation between CEA and tumorigenesis is still unclear; thus, the lack of antitumor potency of anti‐CEA antibodies could be a potential problem. Currently, three antibodies are in clinical trials and all antibodies exhibit enhanced therapeutic potency by new technologies, such as ADC, bispecific antibody, and cytokine fusion antibody 7, 28. The addition of new technology to improve antibody efficacy is an important advancement for the potential therapeutic use of anti‐CEA antibodies; however, highly secreted soluble CEA is also thought to be a hindrance to their use. In this study, we successfully created 15‐1‐32, a fully human anti‐CEA antibody, by immunization of KM mice 3. 15‐1‐32 showed the strongest binding activity to membrane‐bound CEA compared to other existing CEA antibodies, whereas its reactivity to soluble CEA was weak. Thus, 15‐1‐32 was retained on the cell surface, at a level dependent on the membrane‐bound CEA expression level, in the presence of soluble CEA (Fig. 6).

To further investigate the mechanism responsible for this unique binding property of 15‐1‐32 to soluble CEA, binding kinetics parameters were measured using Biacore biosensor. The kinetics analysis revealed that kass of 15‐1‐32 is 10 times higher than that of the existing anti‐CEA antibody, labetuzumab. In contrast, kdiss is only 2.5 times higher than labetuzumab. This suggested that 15‐1‐32 is highly reactive to, but easily removable from, soluble CEA. Although the kinetics analysis of 15‐1‐32 to membrane‐bound CEA was not investigated owing to technical difficulty, we speculate that 15‐1‐32 would retain the high kass, even to the CEA expressed on the cell surface, and that kdiss may decrease, indicating that it is not easily removed from cells. This hypothesis could explain 15‐1‐32's reactivity, that is, its strong binding activity to the cell surface membrane‐bound CEA and weak binding to soluble CEA. In this study, we could not measure the correct kinetics parameters of PR1A3 because of the lower kass; this suggested both PR1A3 and 15‐1‐32 are poorly reactive to soluble CEA, even though the binding mechanism is completely different. To further clarify the binding mechanism, detailed epitope analysis may be needed.

To confirm the tumor tissue binding of 15‐1‐32, we conducted an immunohistochemistry analysis of 15‐1‐32 and labetuzumab binding in human stomach and esophagus cancer tissue. Positive staining was observed in all stomach cancer tissue samples and in six of 10 esophageal cancer tissue samples. Furthermore, there were similar staining patterns between 15‐1‐32 and labetuzumab. This suggests that 15‐1‐32 retains its membrane‐bound CEA recognition property against patient cancer tissues and can be applied for future clinical use.

In this study, we generated a 15‐1‐32 based ADC, 15‐1‐32‐vcMMAE, using our Actibody technology 23, and compared its cytotoxicity to CEA‐expressing MKN‐45 cells with that of labetuzumab‐vcMMAE. The results showed that the cytotoxicity of 15‐1‐32‐vcMMAE was almost the same as the cytotoxicity of labetuzumab‐vcMMAE. CEA has been generally reported as a noninternalizing antigen dependent on antibody binding 29, 30, 31, but many reports have also suggested that anti‐CEA antibodies are internalized due to membrane turnover 32, 33, 34. Furthermore, uptake rates of anti‐CEA antibodies were generally similar, independent of their stability against protease digestion and valency 33. Thus, CEA antibodies might be viable for carrying cytotoxic molecules to tumor tissue for cancer therapy. In fact, the anti‐CEA ADC, labetuzumab‐SN‐38, showed therapeutic effect in a preclinical xenograft model and is now in phase II clinical trials 35.

Additionally, we evaluated these ADC activities against tumor cells in the presence of soluble CEA. The cytotoxicity of labetuzumab‐vcMMAE was inhibited in the presence of soluble CEA, suggesting a strong coupling between soluble CEA and antibody interfered with the binding of labetuzumab‐vcMMAE to membrane‐bound CEA. On the other hand, soluble CEA did not have much influence on the cytotoxicity of 15‐1‐32‐vcMMAE because of its unique binding properties to soluble and membrane‐bound CEA. These results indicate that 15‐1‐32 may be more suitable for antibody‐based drugs, especially antibody drug conjugate, than labetuzumab. To further clarify the potential of 15‐1‐32 in ADC, in vivo analysis of these antibodies, including pharmacokinetics and pharmacodynamics study in the presence of soluble CEA, are required.

In conclusion, we produced and characterized a novel anti‐CEA antibody, 15‐1‐32. Favorable binding properties against membrane‐bound CEA and powerful antitumor effect of vcMMAE conjugate in vitro suggest that 15‐1‐32 has great clinical potential for the detection or treatment of CEA‐positive tumors.

## Conflict of Interest

All of the authors are employees of Kyowa Hakko Kirin Co., Ltd.

## Supporting information


**Figure S1.** Typical sensorgrams of (A) 15‐1‐32 and (B) labetuzumab. Each legend indicated the concentration of soluble CEA.
