# Supplementary material for: Novel anticarcinoembryonic antigen antibody–drug conjugate has antitumor activity in the existence of soluble antigen
Source: Cancer Med. 2017 Feb 17;6(4):798–808. doi: 10.1002/cam4.1003 (PMC5387159; doi:10.1002/cam4.1003)
Supplement: Supplementary file 1 — Figure S1. Typical sensorgrams of (A) 15‐1‐32 and (B) labetuzumab. Each legend indicated the concentration of soluble CEA. [file CAM4-6-798-s001.doc]

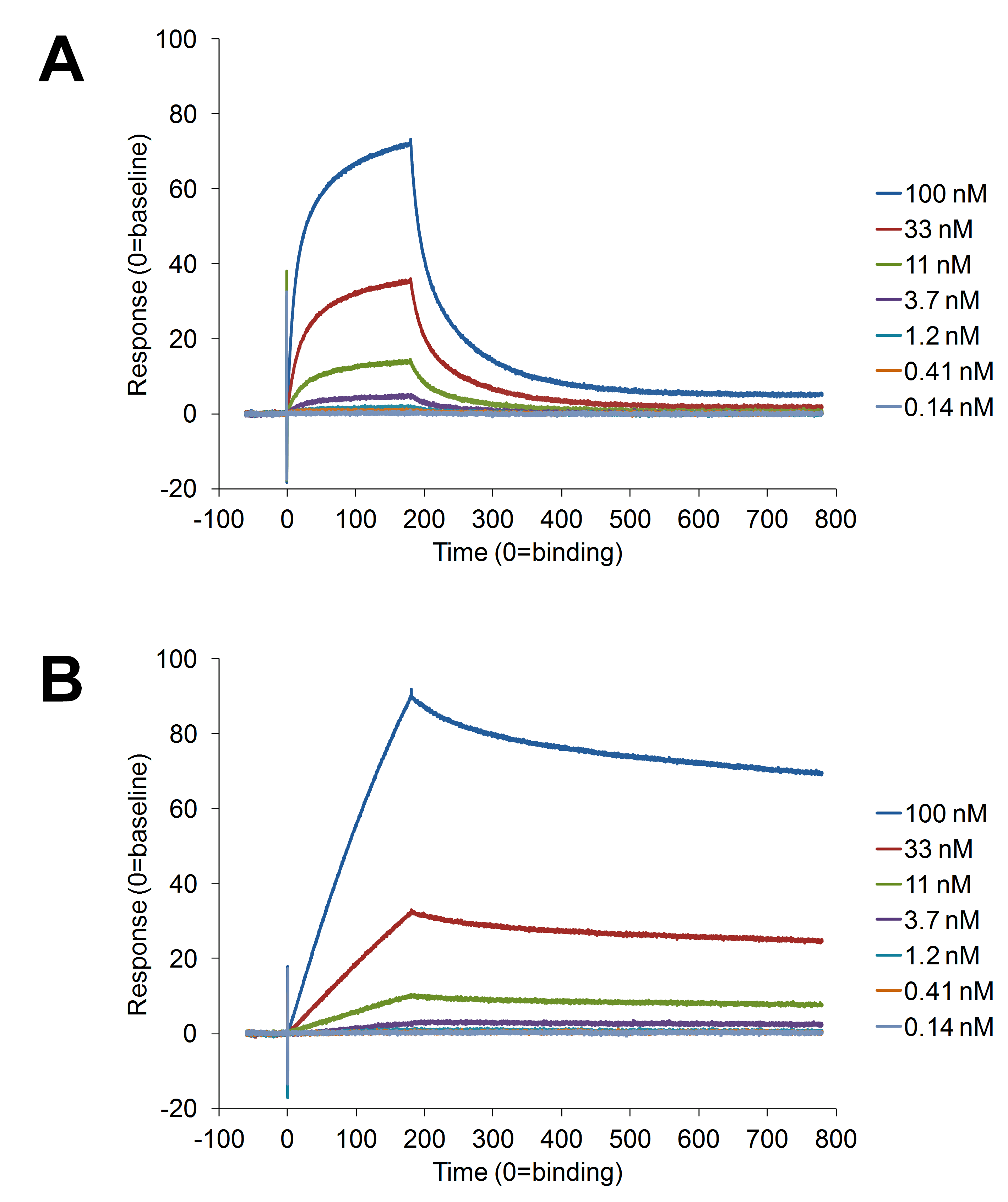


**Figure S1.** Typical sensorgrams of (A) 15-1-32 and (B) labetuzumab. Each legend indicated the concentration of soluble CEA.
